# A Wireless Sensor Network Deployment for Rural and Forest Fire Detection and Verification

**DOI:** 10.3390/s91108722

**Published:** 2009-10-30

**Authors:** Jaime Lloret, Miguel Garcia, Diana Bri, Sandra Sendra

**Affiliations:** Integrated Management Coastal Research Institute, Polytechnic University of Valencia, Camino Vera s/n, 46022, Valencia, Spain; E-Mails: migarpi@posgrado.upv.es (M.G.); diabrmo@posgrado.upv.es (D.B.); sansenco@posgrado.upv.es (S.S.)

**Keywords:** deployment, wireless sensor networks, fire detection, verification

## Abstract

Forest and rural fires are one of the main causes of environmental degradation in Mediterranean countries. Existing fire detection systems only focus on detection, but not on the verification of the fire. However, almost all of them are just simulations, and very few implementations can be found. Besides, the systems in the literature lack scalability. In this paper we show all the steps followed to perform the design, research and development of a wireless multisensor network which mixes sensors with IP cameras in a wireless network in order to detect and verify fire in rural and forest areas of Spain. We have studied how many cameras, sensors and access points are needed to cover a rural or forest area, and the scalability of the system. We have developed a multisensor and when it detects a fire, it sends a sensor alarm through the wireless network to a central server. The central server selects the closest wireless cameras to the multisensor, based on a software application, which are rotated to the sensor that raised the alarm, and sends them a message in order to receive real-time images from the zone. The camera lets the fire fighters corroborate the existence of a fire and avoid false alarms. In this paper, we show the test performance given by a test bench formed by four wireless IP cameras in several situations and the energy consumed when they are transmitting. Moreover, we study the energy consumed by each device when the system is set up. The wireless sensor network could be connected to Internet through a gateway and the images of the cameras could be seen from any part of the world.

## 1. Introduction

When summer comes, the risk of fire is high. Unfortunately, numerous fire foci appear in the Mediterranean zone countries. Hundreds of thousands of hectares are destroyed every year, which produces disastrous environmental, economical, social, material and general infrastructure consequences. Some cases could even cause the death of the inhabitants of the affected zone. Fire entails pollution and water contamination as well as a loss of nutrients and ground microorganisms [1]. Besides, it causes vegetation degradation and flora and fauna diminution because they disappear from the affected zone and are not reintegrated into other environments. The causes that start the forest and rural fires can be classified into six main groups: flashes of lightning, human negligence, fortuitous natural causes, deliberate causes, the reappearance of a previous fire and unknown causes.

Governmental and national authorities, citizens, owners of the lands and the administrations are responsible for taking care of forest and rural places. Spain has a lot of legislation on this matter [2]. New technologies and tools are constantly adapted to the fight against rural and forest fires. Both preventive and post fire detection systems are useful to defend the areas against fire. The fire fighters in charge of the parks and forest zones must have the latest technology and must be equipped to be able to forecast the fire. They must know how it spreads and how to combat it. This is an important issue in order to lower the risk, and to avoid an environmental disaster.

The use of sensors to detect and monitor fire behavior has enhanced the application of new technologies in the fire field. Sensors are able to consider certain dynamic and static variables such as humidity, the type of fuel, slope of the land, the direction and the speed of the wind, smoke, etc. They allow us to determine the direction and possible evolution of the flame front. The sensor-based systems can be very useful to detect a fire and to take decisions to eradicate it.

A sensor is able to transform physical or chemical readings gathered from the environment into signals that can be measured by a system. In our case we have deployed a multisensor node that is able to sense several magnitudes in the same device. In a multisensor, the input variables could be temperature (it is also able to capture quick changes of temperature), fire infrared radiation, humidity, smoke and CO_2_.

A Wireless Sensor Network (WSN) could be a useful architecture for the deployment of the sensors used for fire detection and verification. A WSN consists of many small devices called sensors which measure physical parameters from the environment [3]. The nodes mainly use a broadcast communication and the network topology can change constantly due, for example, to the fact that nodes are prone to fail. They have limited power, computational capabilities and memory. One of the main issues in WSNs is their scalability [4] and their connection strategy for communication [5]. If there is a central server in the sensor network, the sensors can transmit their observations to this central server directly without any processing or they can extract the useful information from their measurements and make decisions to be sent to the central server in distributed detection.

The objective of this paper is to show all the steps followed to perform the design, research and development of an optimized Wireless IP multisensor Network to detect and locate the focus of the fire, and verify it by means of images, and monitor fires in wide extension fields of rural, agricultural and forest using the Wireless Local Area Network (WLAN) technology. First, we studied the number of cameras, multisensors, a device developed by us that is able to sense different type of parameters, and access points that are needed to cover a rural or forest area. We also studied the scalability of the system. The system mixes multisensors with IP cameras in a wireless mesh network in order to detect and verify fire thus minimizing the reaction time of the fire fighters and, therefore, the effects of the fire in rural and forest areas of Spain.

When a fire is detected by a wireless multisensor, the sensor alarm is sent through the wireless network to a central server. The central server runs a software application that selects the closest wireless cameras to the multisensory and gives them coordinates to rotate to the multisensor that raised the alarm, and sends them a message in order to receive real-time images from the zone. It will let the fire fighters corroborate the detected fire. We will also research the power consumption of the devices involved in this deployment in order to demonstrate that it is sustainable.

The paper is organized as follows. Section 2 presents some related works with the use of wireless sensors for fire detection that we have found in the literature. Section 3 describes the main features of a rural area and the research we have done to perform the deployment. The radio design, the analytical considerations to know the number of devices needed, and the channel distribution plan is shown in section 4. Section 5 shows the hardware deployed that has been used in this work. The system design and protocol operation is shown in Section 6. Section 7 shows the user interface for the firefighters. In Section 8, the performance test and the power consumption measurements are presented. Finally, Section 9 shows the conclusion and future works.

## 2. Related Work

Several technological solutions based on wireless networks have been proposed to detect and monitor a fire. The related literature shows systems based on satellites, infrared cameras, wireless cameras and sensor networks. Some of these wireless systems are implemented alone, but there are some that mix several technologies. Moreover, there are other types of technologies, such as a GPS system, which can be added to improve their performance.

There is an important system for forest fire detection based on satellite imagery: MODIS [6]. It studies the images taken from satellites. But, weather conditions are an important problem in these systems. Clouds and rain absorb parts of the frequency spectrum and reduce spectral resolution of satellite imagery. So, the performance of this system changes very much. Satellites can monitor a large area, but the resolution of satellite imagery is low. A fire is detected when it has grown quite a lot, so real time detection cannot be provided. Moreover, these systems are very expensive.

Li *et al.* presented an algorithm based on satellite remote sensing to detect fire across the Canadian boreal forest zone [7]. The authors use images provided by the Advanced Very High Resolution Radiometer (AVHRR). The paper shows the analysis and how their algorithm works in order to detect a fire by using several graphics. The system presents several advantages: automatic operation, consistent data quality, cost-effective use, and rapid response, but not in real-time.

Thierry Antoine-Santoni *et al.* [10] designed a system, called Firesensorsock, to protect every sensor node (mote) of a wireless sensor network in order to avoid these devices being damaged or destroyed when they are sending data, detecting or controlling a fire. Firesensorsock is a special protection dedicated to the thermal insulation of the sensors that leave intact their ability to sense thermal data. Thus, the objective of this work is to have a wireless sensor network that is able to resist being burnt. The sensors will continue transmitting data flow to the final user. Results show a significant change of the temperature and humidity inside the protection, which determines the presence of a fire. Besides, the authors point out that a WSN protected with Firesensorsock is capable of sensing thermal data in the open air. They are able to detect a fire and track the fire spread during its spatial and temporal evolution.

Nowadays, wireless sensor networks are widely used to monitor and to detect a fire, and there is a fair amount of literature on it. An example is the FireBug system. In [8], the authors present a system based on a wireless sensor network for forest fire monitoring. The design is performed with MICA motes using GPS attached. Its objective is to gather environment parameters like temperature, relative humidity and barometric pressure when there is an active fire. Motes communicate with a base station and data are stored in a database server. In order to access to this server, a web browser based on a web application, or any other application capable of communicating with the database server, is necessary. This system uses the Crossbow MICA2 mote and TinyOS programmed in the nesC language. This software is specifically developed for embedded devices. This architecture was tested using 10 motes in two prescribed burns in California on the 16th and 30th of September 2004. Results were satisfactory and motes were capable of reporting data correctly before they were burned.

A proposal for fire rescue applications is described in [9]. First, the authors show the requirements that have to be considered for this kind of network, including accountability of firefighters, real-time monitoring, intelligent scheduling and resource allocation, and web-enabled service and integration. According to these requirements the authors propose FireNet. It is a wireless sensor network architecture where sensors are distributed in the vehicles, forming a self-organized heterogeneous network with the fire fighters. Finally, according to the requirements abovementioned and the characteristics of wireless sensor networks, the authors present several research challenges from the point of view of new protocols, hardware and software for WSNs. FireNet architecture is considered to be very useful in fire rescue processes.

The Forest-fires Surveillance System (FFSS) has been developed to survey the mountains of South Korea [11]. Son *et al.* propose architecture composed of WSNs, a transceiver, middleware and a Web-application. The nodes of this network gather measurements of temperature, humidity and illumination from the environment. These data are concentrated in one node of the WSN called sink-node. This node sends the data to the transceiver (gateway) connected to Internet. Then, a middleware program determines the forest-fire risk-level by a formula from the Forestry Office. If a fire is detected, FFSS automatically activates an alarm to facilitate an early extinguishing of the fire. In this work, the nodes use TinyOs as an operating system. Besides, the WSN use a minimum cost path forwarding (MCF) to send their data to a sink-node.

Hefeeda and Bagheri [12] presented a WSN for forest fire detection based on the FireWeather Index (FWI) System, which is one of the most comprehensive forest fire danger rating systems in North America. This system determines the risk of propagation of a fire according to several index parameters. So, weather data is collected by the sensor nodes and it is analyzed in this work to calculate these indexes. Another aspect analyzed is the number of measurements taken from different sensors to minimize error estimation. They present and simulate a distributed algorithm to solve this problem. Finally, the authors compare their algorithm against others in the literature and they conclude that the proposed algorithm extends the network lifetime and can provide higher detection accuracy in some areas.

The objective of FireWxNet [13] is to determine the behavior of fire rather than its detection. It consists of a WSN that is used to measure weather conditions around an active fire. Webcams are used to get visual data of burned area and a base station which is capable of providing long distance communication. Every half an hour, the system measures temperature, relative humidity, wind speed and direction. In contrast, cameras provide images continuously about the current state of the active fire. The developed system uses five, long-distance wireless links, three sensor networks, and two web-cameras. The results of the system are very good and they show that it is very useful to analyze fire behavior.

To conclude, Garcia *et al.* have presented some papers about sensor networks for fire fighting. In one of them they propose a simulation environment called Equipment Destined for Orientation and Security (EIDOS) [14]. This platform analyzes and combines the geographical information of the area (topography, combustible…) and the data sensed by network nodes (temperature, humidity, wind direction and speed) to create a model of the fire. All these data are sent directly to the firefighters' handheld devices to help them with the forest fire fighting. This paper describes and simulates the proposed system, but it is not implemented in a real environment. The same authors proposed a wireless sensor network to gather environment data in real time [15]. The difference with the other papers is that these data are sent from the wireless sensor network to a base station and they are used to feedback a fire simulator. The approximations calculated by the simulator are more precise and they can be used to compute better predictions about the fire evolution and its behavior.

Systems based on satellite images are not widely used because they do not provide real time fire detection and they are high cost. Nowadays, wireless sensor networks are fashionable in fire-fighting, but although it is the technology most used to detect fires, there are very few implementations published in the literature. Almost all the works published about Wireless Sensor Networks on Rural Fire detection are only theoretical or talk about their possible use, but very few of them present a deployment. On the other hand, in most cases, sensor networks only recollect data about the environment in order to detect and analyze the fire, its behavior and evolution. They do not verify their fire detection.

None of the published systems is like the one presented in this paper. We present the research and design of a system where the fire is detected by wireless IP sensors and the alarm is sent to a central server. The central server selects the closest IP cameras to the fire and lets the firefighter verify an active fire thus decreasing the reaction time.

## 3. Rural Area Features and Topology Design

In this section, we explain the place where we set up our deployment and the main features of a rural area in order to introduce the reader to the main issues that should be taken into account in designing the wireless network. We also study the number of devices needed per coverage area.

**Figure 1.**
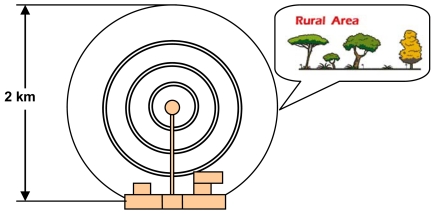
The rural area where the test bench has been performed.

The rural environment, where our test bench is being developed, is a 2 Km diameter circle (see Figure 1). It is located in “El Encín”, Alcalá de Henares, Madrid, Spain. “Explora El Encín” is a popular scientific project of the “Instituto Madrileño de Investigación y Desarrollo Rural, Agrario y Alimentario (IMIDRA)”. IMIDRA is entirely dedicated to research, innovation and scientific spreading tasks. One of the main objectives is to present the surroundings closest to Madrid, its agriculture and its researches to the citizens. It is uninhabited. There are different types of cereal cultivation (wheat, barley, oats, maize, etc.), vegetables (chick-peas, lentils, peas, etc.), grapevines and other types of plants, with different production systems. The area also has different types of trees, with a great fauna and flora variety. The main focus of “Explora el Encín” is to show the natural environment to people who have some physical or sensorial disability. Current society feels more and more that this type of natural space should be protected in order to conserve many species of animals and native plants. Because of the environmental importance, and the great variety of species cultivated inside, we were required to deploy a system to detect a fire, using wireless multisensors, and verify it, using wireless cameras, in order to decrease the reaction time and to avoid a big disaster.

A rural environment video-surveillance design is very different compared to home or enterprise designs. The presence of animals and the reduction in coverage because of the vegetation has to be considered. It involves the following issues:

- We have to minimize the visual impact of the data network in the rural environment. So, data wires should be avoided.
- We have to avoid the use of electric wires because it could damage animals, so the power has to be obtained using batteries and solar panels. It implies that the devices have to be low power consumption to minimize costs and visual impact (the greater the power consumption, the bigger the solar panel).
- The video camera has to be very small in order to reduce the visual impact to animals, but it should have enough quality to obtain good images.
- Enough bandwidth is needed in the wireless network to be able to stream video from different video camera devices.
- The rural area has plenty of trees, animals and vegetation. These objects diminish the received power, so we must be sure that the received signal in our wireless network has enough power.
- Nowadays an 802.11g WLAN has a maximum bandwidth rate of 54 MBps (close to 30 Mbps of effective bandwidth), so we should test how many wireless cameras and multisensors could transmit to a single access point without having a video quality reduction.

**Figure 2.**
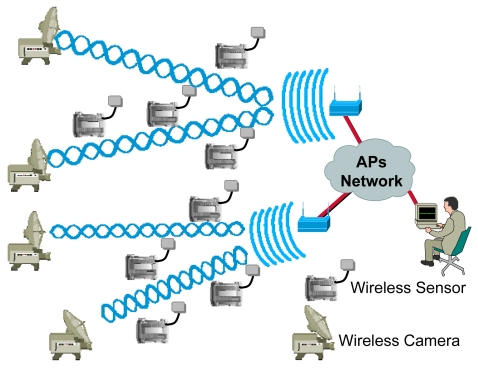
Fire detection and verification design proposal.

Our design uses one or several 802.11g access points (depending on the number of wireless cameras and the number of fire detectors) placed on a visible position from all parts of the rural area. We use wireless IP cameras with high gain antenna (to reach large distances). The wireless multisensors are distributed strategically around the rural or forest area, but always located inside the coverage area of the wireless access points (see Figure 2). Both, multisensors and cameras are under the coverage area of an access points. The access points of the network are connected wirelessly using IEEE 802.11g (if they are close enough) or using optic fiber using IEEE 802.3u. The access points of the network allow the connectivity of all multisensors and IP cameras in the network to a central server. The frames are sent through the data link later without the need of any routing protocol in the network. The position of the multisensors is initially saved in a central server, but it could be changed at any time. Although in our initial implementation the multisensors are not mobile, we can implement mobile sensors that can be monitored using GPS or wireless positioning based systems [16].

The wireless multisensor sends a fire alarm through the access points to a central server if the combination of its physical sensors gives that there is fire. The input variables of the multisensor are fire infrared radiation and smoke, but we are planning to add temperature (and the quick changes of temperature), humidity and CO_2_ [17]. The field of vision of the wireless cameras covers the whole geographical area where the multisensors are placed (the cameras can rotate 270° horizontally and 90° vertically). The central server has a database that relates each multisensor with a certain horizontal and vertical direction of any one of the closest wireless IP cameras. Then, the server sends a frame to the closest cameras. They move their objective toward the multisensor that detected the fire. The images are seen from all cameras placed near the affected zone to a computer placed in the firefighter control room. All devices have an IP address and these images and information could be accessed from Internet.

Because the data registered by the sensors are combined with the decisions of the firefighters after they have seen the images taken from of the cameras, this system offers full information to the firefighter squads, facilitating the extinction tasks. Moreover, the affected area can also be visualized using the controls of the camera at the time. It can also be used to improve fire control and surveillance.

## 4. Radio Design, Number of Devices Needed and Channel Distribution Plan

In order to design the wireless sensor network we have studied the signal loss during its path in a rural or forest environment. We need to know how far the Wireless IP camera and the wireless sensor could be from the access point to receive enough signal power. To calculate this parameter we use the power balance formula (given by equation 1). This equation states that the received signal power, in dBm, is equal to the transmitted power plus the transmitter and receiver gain, minus the basic loss and minus other losses produced by objects (such as trees or humidity) [18].

(1) $$P_{rx}(\text{dBm})=P_{tx}(\text{dBm})+G_{tx}(dB)+G_{rx}(dB)-10\cdot n\cdot \log d-L_{\text{rain}}(dB)-L_{\text{vegetation}}(dB)$$

Where n is the attenuation variation index. *n*=2 for air medium and *d* is the distance between the transmitter and the receiver. We have considered rain loss, which depends on the place where the wireless system is installed, and vegetation loss that depends on the number of trees closer to the signal path between the transmitter and the receiver. None of the works that we have found in the literature about WLAN design have taken into account all the parameters that we have considered in our study [19-21]. The value of these losses can be obtained from references [22] and [23]. So, in our environment, the coverage distance is given by equation 2. More details about the steps followed are given in a previous work from the same authors [24]

(2) $$d=10^{\frac{P_{tx}+G_{tx}+G_{rx}-L_{\text{rain}}-L_{\text{vegetation}}-P_{rx}}{20}}$$

In order to calculate the distance between devices, but bearing in mind the vegetation, we are going to fix some parameters. On the one hand, theoretical transmitted power is –40.2 dBm for an 802.11 g WLAN device at 1 meter, and we estimate -80 dBm threshold power for the far-away IP camera and sensor to have enough quality of signal, so our received power must be greater than or equal to this mark. Let's use a 20 dBi omnidirectional antenna for the access point (*Gtx*), 12 dBi directional yagi antennas for all wireless IP cameras (*Grx_camera_*) and 7 dBi onmidirectional antennas for the sensors (*Grx_sensor_*). On the other hand, this study has been done in Spain, which has two main hydrometric areas: the H area and the K area [25], so losses due to rain, in the worst case, have a value of 0,026 dB for two kilometers. In order to know the losses because of the vegetation, we have used the recommendation given in reference [26], so we can assume a loss of 1.2 dB/m. Equation 3 shows the formula needed to design our WLAN:

(3) $$d=10^{\frac{59.77+\text{Grx}-1.2\cdot m}{20}}$$

**Figure 3.**
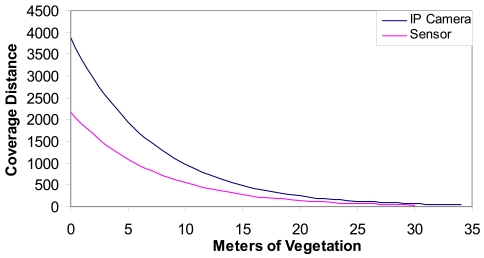
Coverage distance vs. meters of vegetation.

Where *m* is the number of meters of vegetation and *Grx* is the gain of the antenna of the device (the IP camera or the sensor). Figure 3 shows the coverage distance (the distance between access points and the wireless IP cameras and the sensors) as a function of the meters of vegetation through the light of sight path. We can see that around 34 meters of vegetation is allowed in the case of an IP camera and approximately 30 meters of vegetation for the sensors.

The radio coverage of the devices depends on the leafiness of the forest. In order to know the number of devices needed for a given area, we have studied the mean coverage area of every device used in our system. The forest density can vary from 40.000 to 200.000 trees per square kilometer (it means a tree every 50 meters in the first case and a tree every 5 meters in the second case). On the one hand, let us suppose that we are measuring a forest where the trees have an average diameter of 3 meters at a height of 3 meters from the ground (the place where the sensors are placed). We also suppose that a regular forest in Spain has a tree every 10 meters. On the other hand, let us suppose that there are no more than 3 meters of vegetation from the IP camera and the Access Point (because the IP cameras have to be placed very high to acquire a good view from the forest and the access points should be placed strategically in the line of sight of the sensors and the IP cameras), so every camera covers a radius of 2,940 meters approximately.

**Figure 4.**
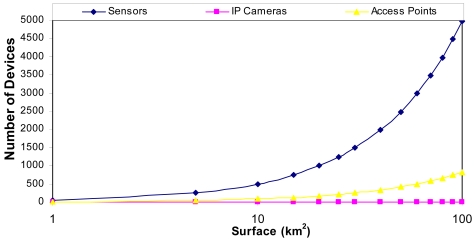
Number of devices needed per area that is wanted to be covered.

Taking into account the measurements provided in Figure 3, if we have 24 meters of vegetation, there could be a distance of 80 meters approximately (a tree every 10 meters with a diameter 3 meters) from the sensor to the access point. Figure 4 shows the number of devices that can be placed in our system as a function to the area that is wanted to be covered. We have supposed the worst case: an access point every 6 sensors in order to maximize the area covered.

Access points are non-root bridge with clients that let the multisensor connect to the access point while it can be connected with root access points. There are access points acting and root bridges that let non-root bridges connect to the infrastructure. The access points that let us configure this type of infrastructure are Cisco Aironet^©^ 350 Series Wireless Bridges [27].

**Figure 5.**
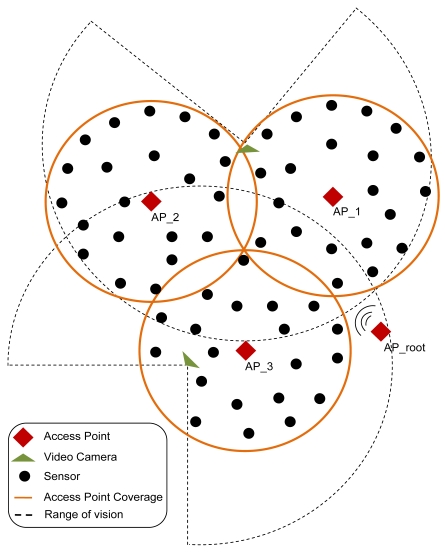
Number of devices needed per area that is wanted to be covered.

Figure 5 shows an example of the topology. There are more sensors than access points and fewer cameras than the other devices (the cameras are the most expensive devices). There is an access point configured as a root and three access points configured as a non-root access points.

The 802.11g standard (in ETSI countries such as Spain) provides 13 channels inside the Industrial, Scientific and Medical (ISM) band, which belong to 13 frequencies between 2412 MHz and 2472 MHz as it is shown in Table 1.

However, the spectrum width used by each channel is overlapped by the adjacent channels, causing interferences. These interferences are higher in closer channels. Table 2 shows the level of interference classified in three levels. Concerning Table 2, in order to select the maximum number of simultaneous channels without any interference, channels 1–5–9–13 must be used. However, when there is a slight interference that does not degrade the system, in practice, the use of channels 1–4–7–10–13 is tolerated. This second option provides one usable channel more, five in total. To be able to reuse these channels, we would have to go far away enough to have no interference.

## 5. Hardware Deployment

In this section we show a sensor node that is able to sense several parameters from the same place while it is able to form an IP network of multisensors. In order to achieve our aim, we looked for a device with a control unit. This control unit manages and controls all sensors connected to the device. On the other hand, the electronic circuit must have several input interfaces in order to connect several physical sensors. Several of the main aspects taken into account were circuit costs, the operative system used and the possibility of adding several physical sensors to the device, in order to enable optimum choice.

### 5.1. Wireless Sensor

**Figure 6.**
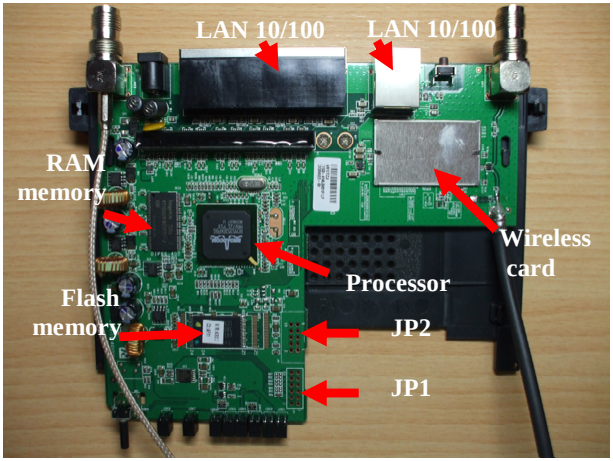
Hardware distribution on board.

Our proposal is based on the use of the Linksys WRT54GL router, from Cisco Systems inc., as the core controller [28]. It is an embedded system that has a wireless IEEE 802.11 b/g interface, a FastEthernet interface in its board, so it meets our pre-requisites. In addition, Linksys WRT54GL offers internally General Purpose Input/Output (GPIO), UART (JP2) and ETAJ (JP1) ports. Some extensions can be made to the router by using these ports. Figure 6 shows the embedded board and its hardware distribution.

One of the main features that have caused the use of the Linksys WRT54GL as a sensor node was the possibility of installing a Linux Kernel 2.4. On one hand, it is a known operative system, so we did not need to learn a new operative system and, on the other hand, we know all the possibilities that a Linux is able to provide us. So, at software level, this model is based on open source software, causing the development of different specific software applications for it and expanding the factory default capabilities.

**Figure 7.**
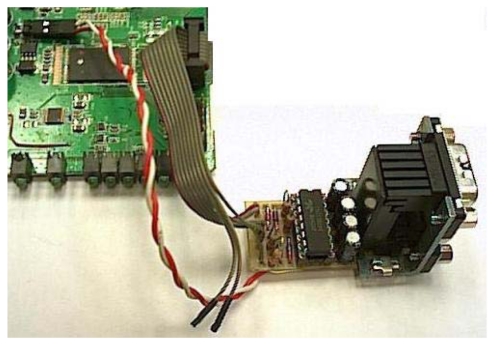
Integrated Circuit used to provide two serial ports.

In order to connect two sensors directly to the board, we made an extension using the GPIO of the Linksys WRT54GL router. It provided us two serial ports through the JP2 port. Figure 7 shows serial ports connected on board by welding pins on JP2. Then we added two DB9 Female DCE ports because we wanted flexibility in order to change the type of sensor connected to our device. To be able to connect a device with RS232 connection, it is needed a logical levels adapter based on MAX233 Integrated Circuit. The Linksys WRT54GL router has TTL family logical levels so we had to adapt them to the RS232 family logical levels. Figure 7 shows the integrated circuit used to provide two serial ports. A RS232 line converter is needed to go from +3.3V to +5V.

Also, we can connect an SD card reader to some of the GPIO pins found inside the Linksys WRT54GL router and with the help of a little driver we can use as a block device from Linux. The SD card reader has been tested for a 1GB SD card. It allows us to install applications for signal processing and store and manage acquired data from both sensors. Now, the sensor node is able to store and process data without the need of sending the measurements taken continuously. We developed a process that is running in the Linksys WRT54GL router. It checks the values obtained by both serial ports. In one port we connected a smoke detector and in the other port we connected a fire infrared detector. The input variables of the multisensor are fire infrared radiation and smoke, but we are planning to add temperature (and the quick changes of temperature), humidity and CO_2_. We programmed a software application that only gives a positive value if both sensors have values higher than a threshold; otherwise it is a false alarm. But this decision can be changed as desired using a combination of the measurements gathered. The system is able to gather data, process it internally, and send only alarms or statistical data spontaneously, saving energy.

### 5.2. Wireless IP Camera

The wireless cameras selected transmit MPEG-4 standard video compression, which has higher compression and quality compared to other standards. It also consumes low bandwidth. MPEG-4 is commonly used in video streaming over IP environments. The video is streamed using the HTTP protocol (we have chosen this protocol to facilitate the video visualization) with very good results. Chosen cameras are able to stream video with a resolution of 320 × 240 using 25 fps (PAL system) and they are able to transmit audio in both directions (from the camera and to the camera). Their working temperature is between −5.5°C and 75°C.

Wireless camera video streaming is transmitted from the camera to the server directly. The person sited in the server (a fire fighter) can choose by software the camera to be watched and the software opens a connection with that camera and receives the video streams sent via the camera.

### 5.3. Photovoltaic System

The photovoltaic system is formed by a photovoltaic panel, the battery, the load regulator and an inverter. There are 3 basic types of photovoltaic panels, all of them use silicon: monocrystalline cells, polycrystalline cells and amorphous cells. We have used the polycrystalline cells because they have higher performance than the amorphous cells (between 11% and 13%) and they are cheaper than the monocrystalline cells. There are many types of batteries that can be used in a photovoltaic system: Lead-acid battery, VRLA battery, AGM battery, Gel battery, Nickel-cadmium battery, etc.

First we studied the solar radiation map of Spain. Taking into account the values provided by references [29] and [30], Spain has high solar radiation values. Concretely, Madrid, the place where we deployed our test bench, has 1,560 hours of sunlight per year, which means a mean value of 4.27 hours of sunlight per day. The battery of the sensor has to be able to provide power during 20 hours at least.

In our case, the sensor consumes 12V and 100 mA approximately, so the power consumed by the sensor is 1.2 W. Taking into account that a polycrystalline cell has a mean performance of 12%, the power needed to supply the sensor and to charge the battery simultaneously is 60 W. Let us suppose that we use have a battery of 24VAh fully charged. The sensor will discharge the battery in 240 hours if there is not sunlight. Such number of hours without sunlight is very difficult to happen in Spain.

## 6. System Design and Operation Mode

First, we placed the wireless IP cameras in strategic places to watch interesting zones. Then, we placed wireless IP sensors in some critical points with more risk. Both, sensors and cameras are under the coverage area of the access points.

The mode of operation is as follows. All cameras have been recorded with the coordinates where they have to move and focus for each sensor placed in their visual coverage. The server has a database with the position of the sensors and the name of the cameras placed in the rural area close to every sensor (they are stored by name: sensor_1, sensor_2, etc.). When a sensor detects a fire, it sends an alarm directly to the server. This alarm message has the name of the sensor. When the server receives this message, it searches in its database the closest wireless cameras to that sensor and sends them a message with the name of the sensor that has sent the alarm and the position they must move to in order to watch the image of that zone. Finally, the cameras move their objective to the coordinates of the sensor. The camera will show what is happening in that zone and the fire fighter can corroborate if there is a fire or not.

**Figure 8.**
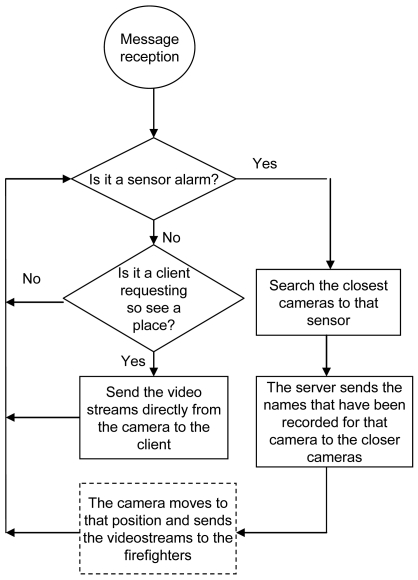
System Mode Operation Flowchart.

When there is an alarm, a firefighter sees the video streams from all the wireless IP cameras of the affected zone. On the other hand, the images could also be watched by other users when they request it. Figure 8 shows the flowchart with the mode operation from the point of view of the system.

**Figure 9.**
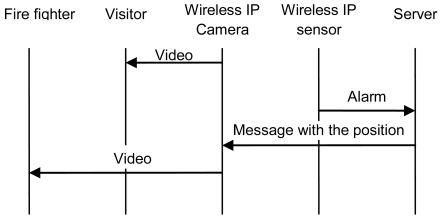
Messages when there is an alarm.

The system is highly scalable because a camera can cover as many sensors as positions can be recorded. On one hand, if it is placed at the top of a mountain, more area can be viewed by the camera. On the other hand, the database of the server can have many entries. There has to be one entry for each sensor. In our design plan we have considered that every sensor has to be seen by two wireless IP cameras at least. We have programmed all these instructions over http protocol to be easily implemented in other systems. Figure 9 shows the messages sent when there is an alarm.

## 7. User Interface

**Figure 10.**
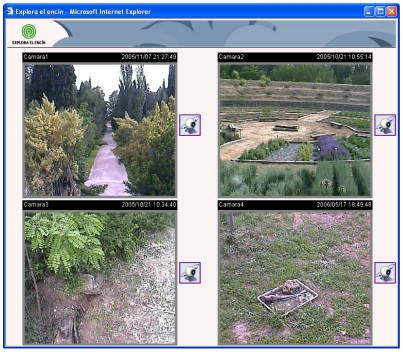
Main web page.

We have developed a web page that shows the video streams received from several Wireless IP cameras in real time. Images are shown without jumps and there is not any quality images reduction. Figure 10 shows the developed web page.

**Figure 11.**
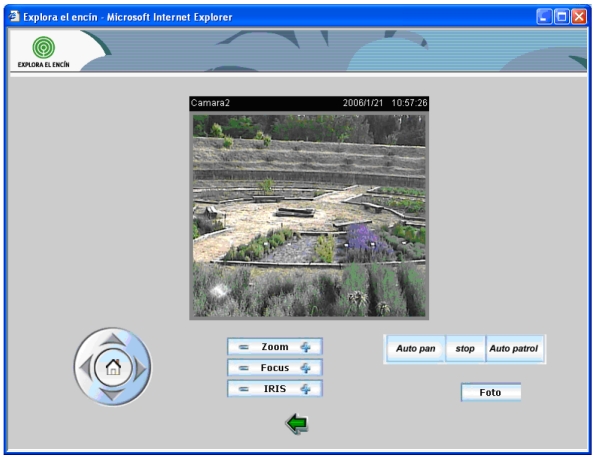
A Single Camera visualization web page.

Clicking the icons placed on the right of every video image, we can access the control web page for each camera. The web page for each camera (see Figure 11) shows a greater image with the same quality and the user is able to change its vertical (till 90 degrees) and horizontal (till 270 degrees) orientation.

We can also vary the zoom lens to a 10× value and focus the video image (it can be done automatically). We can vary the Iris lens to obtain a better visualization and we have enabled two buttons called “Auto pan” and “Auto patrol” that moves the camera automatically to have a panoramic view of the place. We have also enabled a button to pick up photographs.

All cameras can be accessed independently and their control is independent, so users or the fire fighter can access to a camera and control it without any disturbance.

## 8. Performance Test

This section shows several test benches that have been set up in order to show the performance of the implementation.

### 8.1. IP Camera Bandwidth Consumption Comparison

We have measured a wireless IP camera placed in the rural area transmitting MPEG-4 codec compressed video over http protocol in order to test the number of bytes per second and the number of messages per second in the network. It lets us know the camera bandwidth consumption. The video resolution was 320 × 240 at 25 fps. The wireless IP camera also transmitted audio at 24 Kbps from the camera to the web page. We have measured, for 2 minutes and 30 seconds, the following situations:

- 1st Situation: The camera is acquiring video from a fixed place where there is not any motion, and the camera is not moving.
- 2nd Situation: The camera is acquiring video from a fixed place where there is motion, but the camera is not moving.
- 3rd Situation: The camera is acquiring video while it is moving.
- 4th Situation: There are 4 cameras acquiring video from fixed places where there is not any motion and the cameras are not moving. We show how much bandwidth just one camera needs.

Then, we have measured the network for 15 minutes in order to know how the network performs for longer times.

**Figure 12.**
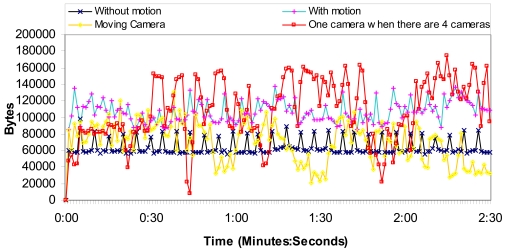
Octets per second obtained by the four situations.

Figure 12 shows the number of bytes per second for the 1st, 2nd, 3rd and 4th situations. We can observe that the worst case is when the video acquired has motion. On the other hand, we think that the moving camera situation is rampant because of the bandwidth used to control the camera. There is no case with more than 140,000 bytes per second (1.12 Mbps). Then, we have divided the number of bytes per second obtained by the number of cameras. The average number of bytes is around 109.2 KBytes (873 Kbits), while the average number of bytes per second when there is just one camera is 63.6 KBytes (509 Kbits). So the system allows 34 cameras in each access point.

**Figure 13.**
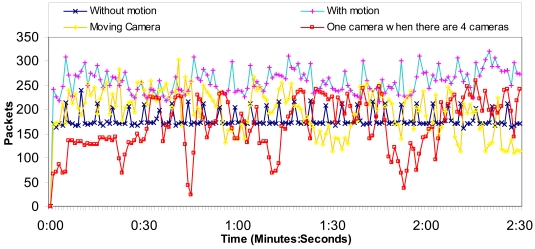
Packets per second obtained by the four situations.

Figure 13 shows the number of packets per second for the 1st, 2nd, 3rd and 4th situations. We can observe that the worst case is when the video acquired has motion. The moving camera sends messages in a rampant manner without any rule. The average number of packets per second, due to one camera, when there are four cameras is around 167 packets/s, while the average number of packets per second when there is just one camera is 180 packets/s. So the system allows many cameras.

**Figure 14.**
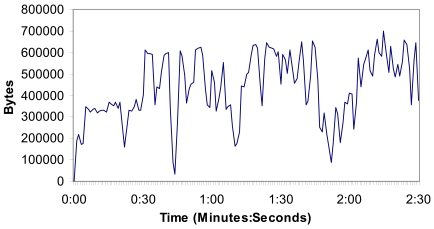
Bytes per second when there are four cameras.

Figure 14 shows the number of bytes per second when there are four cameras (in the 4^th^ situation, but the measurements of all cameras). We can observe that there is not more than 700.000 bytes per second (5.6 Mbps) in the wireless network. Measurements show us that the implemented system can support video streaming from four wireless IP cameras without problems.

**Figure 15.**
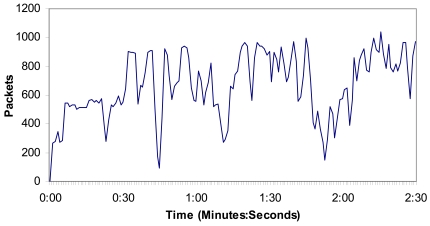
Packets per second when there are four cameras.

Figure 15 shows the number of packets per second in the 4^th^ situation, but showing the measurements of all cameras. We can observe that it is not more than 1000 packets per second in the wireless network, so there are not so many messages through the medium at the same time. As the number of packets sent through the network is proportional to the number of cameras in the wireless network, 34 cameras will send less than 34,000 packets per second through the network.

### 8.2. IP Camera Bandwidth Consumption Performance and Stability

We have measured all four wireless IP cameras transmitting MPEG-4 codec compressed video over HTTP protocol in order to test the number of bytes per second and the number of messages per second through the network during 15 minutes in order to see the performance of the network and its stability. The video resolution was 320 × 240 at 25 fps. The wireless IP camera also transmitted audio at 24 Kbps from the camera to the web page.

**Figure 16.**
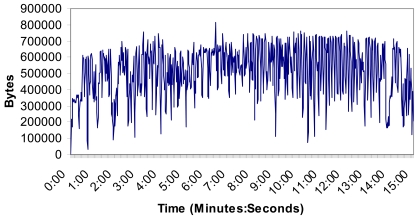
Number of bytes per second.

Figure 16 shows the number of bytes per second. There are not more than 800,000 bytes in the wireless network and the mean value was 514,013 bytes. The measurements show that the implemented system can support video streaming from all four Wireless IP cameras without problems because there is not so much bandwidth consumed and the system is stable.

**Figure 17.**
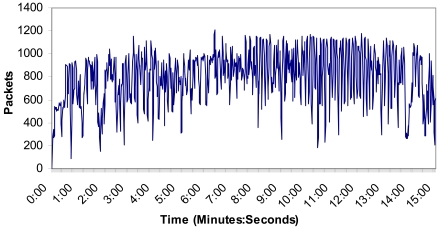
Number of packets per second.

Figure 17 shows the number of packets per second in this case. No packet peak reaches 1,200 packets in the wireless network and the mean value was 798 packets. There are not too many messages through the medium at the same time. As the number of packets sent through the network is proportional to the number of cameras in the wireless network, 34 cameras will send around 40,800 packets per second through the network in this case. On the other hand, we could add one more access point. Taking into account the area covered by the cameras (few cameras are needed to cover large distances) and the number of sensors per each access point (see the research study made in figure 4), the designed system is scalable with the only limitation of the bandwidth of the backbone technology used to join the access points.

### 8.3. Multisensor Bandwidth Consumption

**Figure 18.**
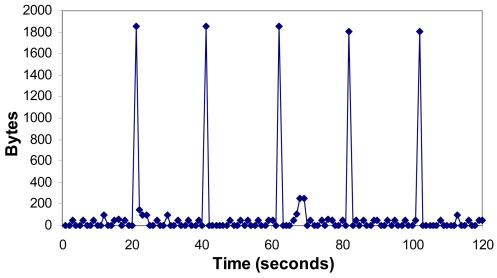
Bytes per second when there is an alarm.

Figure 18 shows the number of bytes per second when a multisensor sends an alarm to the server. Peaks value is 1850 bytes, and it is due to the network traffic. The alarm was sent between the 60 and the 70 seconds, so it consumes very low bandwidth. There is an average value of 100.35 bytes per second because of the multisensors.

**Figure 19.**
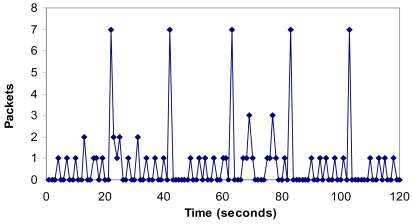
Packets per second when there is an alarm.

Figure 19 shows the number of packets per second when there is an alarm. It shows that the maximum number of packets per second in the network is seven. We have shown that an alarm makes peaks of 3 packets as is shown between the 60th and the 70th second. There is an average value of 0.68 packets per second.

### 8.4. Power Consumption

Although we have provided every device with batteries and solar panels that allow charging the batteries when the sun is shining, we have studied the energy consumption of every device. In this section we provide the power consumption for all devices used in our deployment.

Table 3 shows the power consumption for each device depending on its state. The device that consumes more power is the Cisco Aironet 350 Bridge and the one that consumes less power (but if it is not moving) is the Wireless IP Camera (D-Link DCS-5220). On the other hand, we can observe that the wireless IP Camera does not have “Idle mode”, so it will be sending images all the time. So it has higher power consumption.

**Figure 20.**
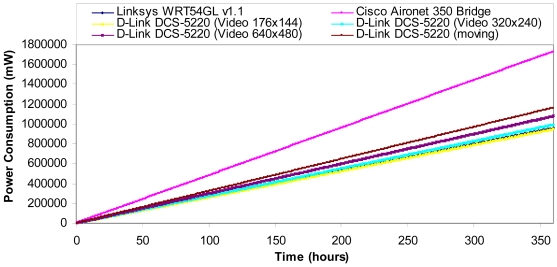
Power consumption.

In order to show the power consumption of the devices shown in table 2 over time, we consider that the circuit used to regulate the battery needed for all the devices consumes the same energy and it is quite low compared with the energy consumed by the devices. The energy consumed by the sensor is also negligible compared with the energy wasted by the chips. Taking into account measurements taken in [31], we suppose that a wireless link spends 70 percent of the time idle (in standby mode), 20 percent of the time receiving, and 10 percent of the time transmitting. Figure 20 shows the power consumption over time by the devices shown in Table 2. The device that consumes more power in our system is the Cisco Aironet © 350 Bridge, which is used as Non-root Access Points and as root Access Points. The worst case for the IP Camera is when it is always moving. It does not happen too much. The best cases are when the multisensor and the IP Camera transmit with a video quality of 176 × 144 at 25 fps.

In order to simulate the total amount of time that a multisensor will be able to be transmitting and receiving with a totally charged battery, we take into account the considerations and values provided in [32] by Heinzelman *et al.* The transmission and the reception energy are given by the expressions 4 and 5:

(4) $$E_{Tx}(k,r)=(E_{Tx}+E_{\text{amp}}r^{2})\cdot k$$

(5) $$E_{Rx}(k)=E_{Rx}\cdot k$$

where *k* is the number of bits sent in the packet, *E_Tx_* = *E_Rx_* = 50 nJ/bit, *E_amp_* = 100 pJ/b/m^2^.

**Figure 21.**
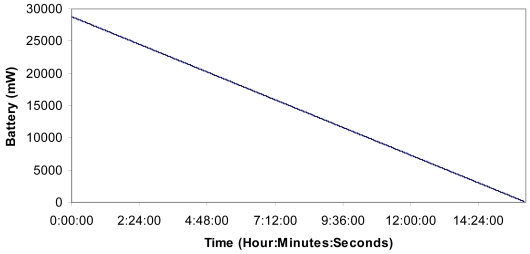
Power consumption in the network because of the packets transmitted and received.

Taking into account the measurements taken in section 8.3, the multisensors send an average value of 1,175 bits per packet. Now, we can estimate the power consumption because of the packets transmitted and received by all multisensors over time. The multisensor is supplied by a 12 V 24 Ah battery with a solar panel, but we switch off the solar panel (no sun shinning for many days) in order to know its lifetime. We have considered the circuit energy consumption, the data processing energy consumption, the energy, when it is reading and writing in the memory, and the energy consumed by the physical sensor as being close to zero because the energy consumed is very little compared with that consumed by the nodes due to all the packets sent and received. Figure 21 shows the energy consumption of a multisensor in the worst case (always transmitting or receiving without any time being in the idle mode). The multisensor will be able to be in this hard mode for more than 16 hours. Then, the battery has to be charged using the solar panel

## 9. Conclusions

We have shown the design, development and the performance test of a Wireless Sensor Network for rural and forest environments fire detection and verification. We have shown the deployment of a multisensor based on a Linksys WRT54GL router that is able to sense fire by infrared radiation and smoke. It is able to send an alarm if the combination of both physical sensors gives as a result that there is a fire. We have studied how many cameras, multisensors and access points are needed to cover a rural or forest area and the scalability of the system. The technology used has been IEEE 802.11g standard. It is flexible and it could be adapted to any type of environment. We have designed it trying to minimize the material cost of its implementation but without diminishing the quality of the video and taking into account the 802.11g WLAN performance. Our design is scalable because we can add access points easily and increment the number of wireless IP cameras attached to these access points. Moreover, it is easy to add emergent Technologies.

When a fire is detected by a wireless IP multisensor, the sensor alarm is sent through the wireless network to a central server. The central server runs a software application that selects the closest wireless IP cameras to the sensor and sends them a message in order to receive real-time images from the affected zone. It lets the fire fighter corroborate the fire by means of a real time visualization of the place where the fire has taken place.

The bandwidth consumption measurements given by our test bench show that the system supports up to 34 wireless IP cameras in each Access Point. We have demonstrated that the control messages developed imply little bandwidth consumption. So, our design is scalable because we can add access points easily and increment the number of cameras and sensors.

We have researched the power consumption of all devices used in our work. We have studied their evolution over time. We have also studied the energy wasted in the network because of the packets transmitted and received.

The router shown can be applied to any environment that needs to be sensed by several types of variables. It is also able to process internally the measurements taken from the connected sensors and send the processed information to a remote site. It is flexible and it could be adapted to any type of environment and to any type of physical sensor with a serial output. Only the programming code needs to be changed to adapt the control management to different sensors.

Now, we are working in several research lines. The first one is to add more input variables in the multisensor. We are planning to add temperature (and the quick changes of temperature), humidity and CO_2_. The second one is to reduce the power consumption of the Linksys WRT54GL router. The third one is focused on adding mobility to the wireless multisensors (by having smaller batteries and solar panels). Finally, the fourth one is to add an algorithm to the system, based on a wireless positioning algorithm [16], to find the place where the multisensors are sensing.

## Figures and Tables

**Table 1.** Channels inside the ISM band.

**Channel**	**Frequency (MHz)**	**Channel**	**Frequency (MHz)**
1	2412	8	2447
2	2417	9	2452
3	2422	10	2457
4	2427	11	2462
5	2432	12	2467
6	2437	13	2472
7	2442		

**Table 2.** An optimal 4-channel distribution over a horizontal area.

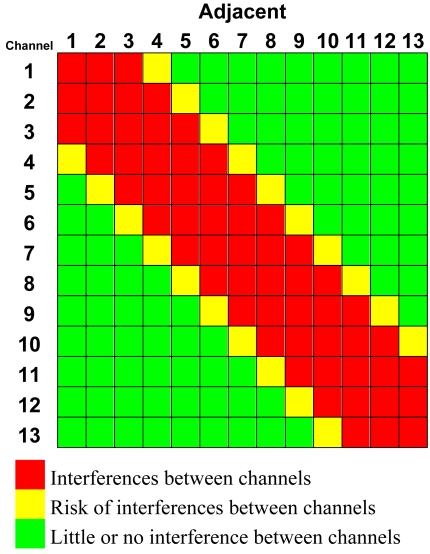

**Table 3.** Devices consumption.

**Device**	**Idle Mode**	**Wireless ON**	**Transmitting or Receiving**
***Linksys*** ***WRT54GL v1.1***	2,400 mW	3,240 mW	3,360 mW
***Cisco Aironet*** ***350 Bridge***	4,320 mW	5,760 mW	6,240 mW
***D-Link*** ***DCS-5220***	–	2,640 mW (video: 176×144)	2,640 mW (video: 176×144)
		2,760 mW (video: 320×240)	2,760 mW (video: 320×240)
		3,000 mW (video: 640×480)	3,000 mW (video: 640×480)
			5,280 mW (moving the camera)
